# Permanent molar pulpotomy with a new endodontic cement: A case series

**DOI:** 10.4103/0972-0707.53340

**Published:** 2009

**Authors:** Saeed Asgary, Sara Ehsani

**Affiliations:** Iranian Center for Endodontic Research, Dental Research Center, Dental School, Tehran, Iran; 1 Dental Research Center, Shahid Beheshti University MC, Tehran, Iran

**Keywords:** Mature, pulpitis, pulpotomy

## Abstract

The aim of this case series was to determine the clinical and radiographic success rate of pulpotomy, with new endodontic cement (NEC), in human mature permanent molar teeth. Twelve molars with established irreversible pulpitis were selected from patients 14 – 62 years old. The selection criteria included carious pulp exposure with a positive history of lingering pain. After isolation, caries removal, and pulp exposure, pulpotomy with NEC was performed and a permanent restoration was immediately placed. At the first recall (+1 day) no patients reported postoperative pain. One wisdom tooth had been extracted after two months because of failure in coronal restoration. Eleven patients were available for the second recall, with a mean time of 15.8 months. Clinical and radiographic examination revealed that all teeth were functional and free of signs and symptoms. Histological examination of the extracted teeth revealed complete dentin bridge formation and a normal pulp. Although the results favored the use of NEC, more studies with larger samples and a longer recall period were suggested, to justify the use of this novel material for treatment of irreversible pulpitis in human permanent molar teeth.

## INTRODUCTION

Since the 1960s, studies have shown that the carious process is initiated by oral bacteria.[1] As the caries progresses within the tooth tissue, the risk of inflammation of the pulp increases and loss of vitality may ensue.[2] Trauma or cavity preparation may also cause pulpal exposure. 

In most cases of pain associated with pulpal exposure two immediate treatment modalities come to mind, root canal therapy (RCT) and extraction. It has been argued that RCT increases susceptibility to fracture due to coronal and apical tooth structure loss.[3] Also it is suggested that after RCT, most teeth should be restored with extensive coronal restoration.[4] The second option, tooth extraction, has its obvious disadvantages, which the patient may have to tolerate for a lifetime if the tooth is not replaced.[5] In such circumstances, the dentist may have the option to maintain pulp vitality by vital pulp therapy or to perform RCT.[6] However, clinicians tend to prescribe pulpectomy even when vital pulp therapy is possible.[7]

Pulpotomy is based on the rationale that after amputation of infected or affected coronal pulp if the remaining pulp can be protected with hard tissue stimulating material, the radicular pulp would heal[8] and the pulp would remain vital.[9] 

Pulpotomy is a universally accepted treatment of teeth with incompletely formed roots, involving pulpal exposure.[9,10] In permanent teeth, it has been postulated that extirpating pulpal tissue in many cases is not cost-effective, but time-consuming, and difficult for both patient and clinician. In addition, failure of vital pulp therapy would not weaken the chances of RCT for the tooth.[10] However, more studies are required to evaluate this procedure in mature permanent teeth.

In 1998, Nosrat and Nosrat[11] performed partial pulpotomy on six permanent molars (four adolescents, 10–15 years of age, and two adults, 20 and 27 years of age) with deep carious lesions and pulpal involvement. After achieving hemostasis they covered the exposed pulp with calcium hydroxide (CH) paste followed by zinc oxide eugenol, and semi-permanent restoration. All teeth demonstrated a hard tissue bridge formation within three months and were asymptomatic. They suggested partial pulpotomy as an alternative treatment for pulpal exposure of deep carious lesions. 

In 2004, McDougal *et al*.,[5] selected 73 patients, 18–65 years of age, with irreversible pulpitis. They restored their teeth after pulpotomy with either Caulk IRM or IRM base with glass ionomer core and followed up the patients for 12 months; assessing pain, integrity of restoration, and densitometric radiographic analysis. They concluded that eugenol pulpotomy prevented pain for six months; however, coronal restoration repair may be required for a longer period.

Nyerere *et al*.,[12] in 2006, selected 180 Tanzanian patients over 15 years with dental pain caused by acute pulpitis. Pulpotomy was performed on the premolars or molars using eugenol. The treatment was evaluated after one, three, and six weeks. RCT and restorations were performed for all cases at appropriate intervals. The success of treatment of the emergency pulpotomy was high (100% for premolars and 97.1% for molars) suggesting this treatment as an alternative for alleviating acute dental pain.

Despite several materials used in these clinical studies, there is as yet no consensus on the ideal pulpotomy agent, even for primary molars.[13] It is universally accepted that the material should be biocompatible, antibacterial, and have an effective seal.[9,14] Previous studies have suggested that the material should stimulate hard tissue regeneration.[9] 

In recent years, mineral trioxide aggregate (MTA) has been introduced for pulpotomy in primary molars.[15] MTA has demonstrated good biocompatibility,[16] excellent sealing ability,[17] and stimulation of healing in the pulpal tissue.[14,16] Dentinal bridge formation was the usual occurrence when MTA was used as a pulp-capping agent in dogs,[16] monkeys,[14] and as a pulpotomy material in mature human teeth.[18] In the first report of MTA pulpotomy in human mature permanent teeth, a case series of 14 human mature permanent molars with irreversible pulpitis, a histological examination revealed complete dentinal bridge formation, pulp vitality, and absence of inflammation in all the cases.[18] However, despite its excellent properties, it has been reported that MTA shows disadvantages including a nonpredictable antimicrobial activity,[19] difficult management, expanded setting time, and a high price.[20,21] However, NEC, a new endodontic material, does not have MTA′s disadvantages[19] and it has demonstrated stimulation of dentinal bridge formation,[16] a sealing ability superior to IRM and comparable to MTA,[22] as well as the ability to set in aqueous environments.[20] Moreover, the antibacterial effect of NEC is comparable to CH and superior to MTA,[19] and its physical properties are better than MTA.[20]

This article describes the clinical and radiographic outcome of cases using NEC in the pulpotomy of human mature permanent molars with caries exposure and established irreversible pulpitis.

## CASE REPORTS

The Ethics Committee of Dental Research Center, Shahid Beheshti University M.C. approved this study. We treated 12 mandibular or maxillary molars (in 12 patients) in an endodontic private clinic. There was no medical contraindication for dental treatment in the subjects. All patients had irreversible pulpitis, moderate-to-severe pain of endodontic origin, complete apical closure, and a vital pulp. Informed consent was signed by all subjects after a thorough explanation of the possible complications of the procedure. 

Teeth were first anesthetized with Lidocaine 2% and adrenalin 1/80000 (Daroupakhsh, Tehran, Iran) and isolated with a rubber dam. The treatment included caries removal followed by pulpotomy, that is, removing the inflamed pulp to orifice level with a large high-speed round diamond bur accompanied with copious irrigation. Associated bleeding in all the cases indicated pulp vitality. Hemostasis was achieved by irrigation with sterile normal saline along with gentle application of small pieces of sterile cotton pellets for five minutes. NEC powder was prepared to a creamy paste consistency. An approximately 2-millimeter-thick layer of NEC was inserted over the exposed clot-free pulpal wound with a plastic instrument and packed with dry cotton pellets. Permanent restoration was placed during the same appointment. 

The patients were recalled after one day for evaluation of postoperative pain. On the next recall session, in addition to radiographic examination, test for percussion sensitivity, soft tissue inspection, and palpation of alveolar areas over the roots were carried out, to reveal any signs of inflammation. 

**Figure 1:**
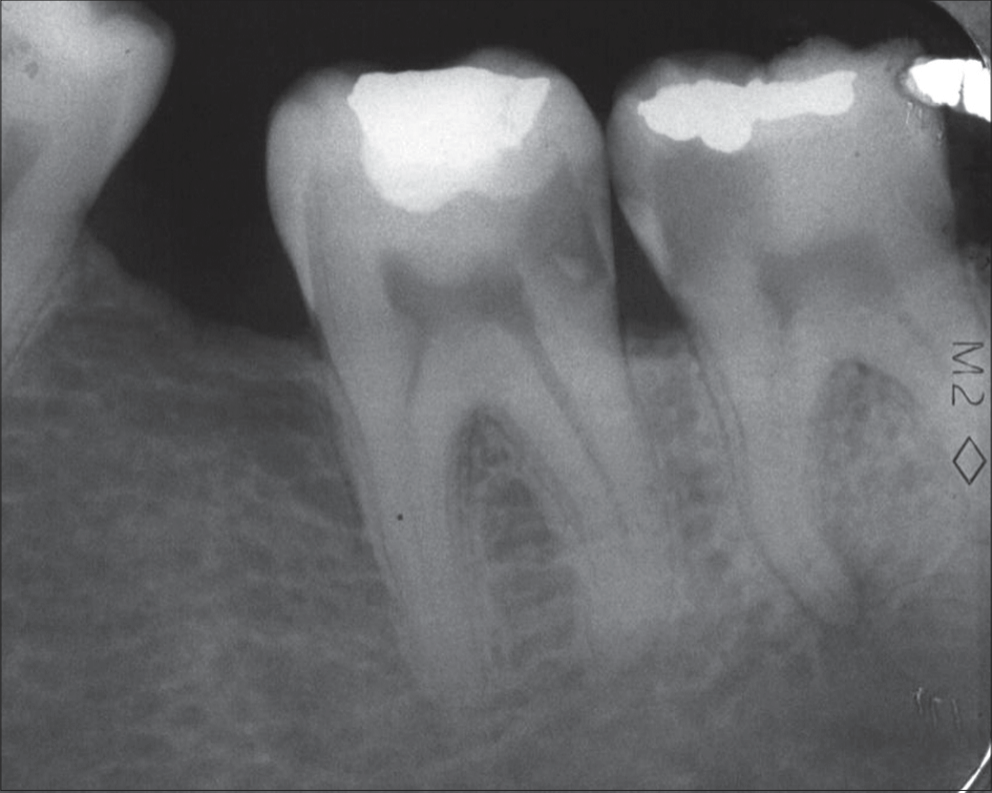
Preoperative radiograph of the second and third lower left molars shows deep interproximal carious lesions. The patient's chief complaint was severe lingering pain with sensitivity to percussion in both involved teeth. A periradicular lesion in the mesial root of the third molar is clear

**Figure 2:**
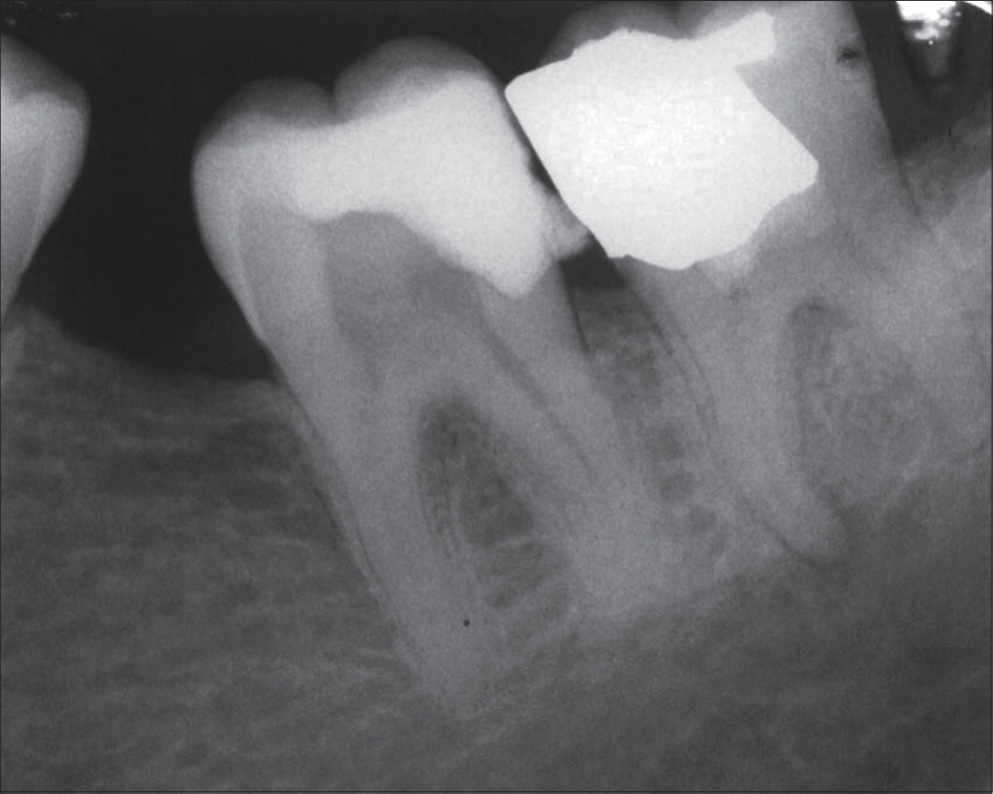
Pulpotomy treatment with new endodontic material was performed on the third lower molar accompanied by permanent restoration. Emergency treatment for the second molar was carried out in the same session

**Figure 3:**
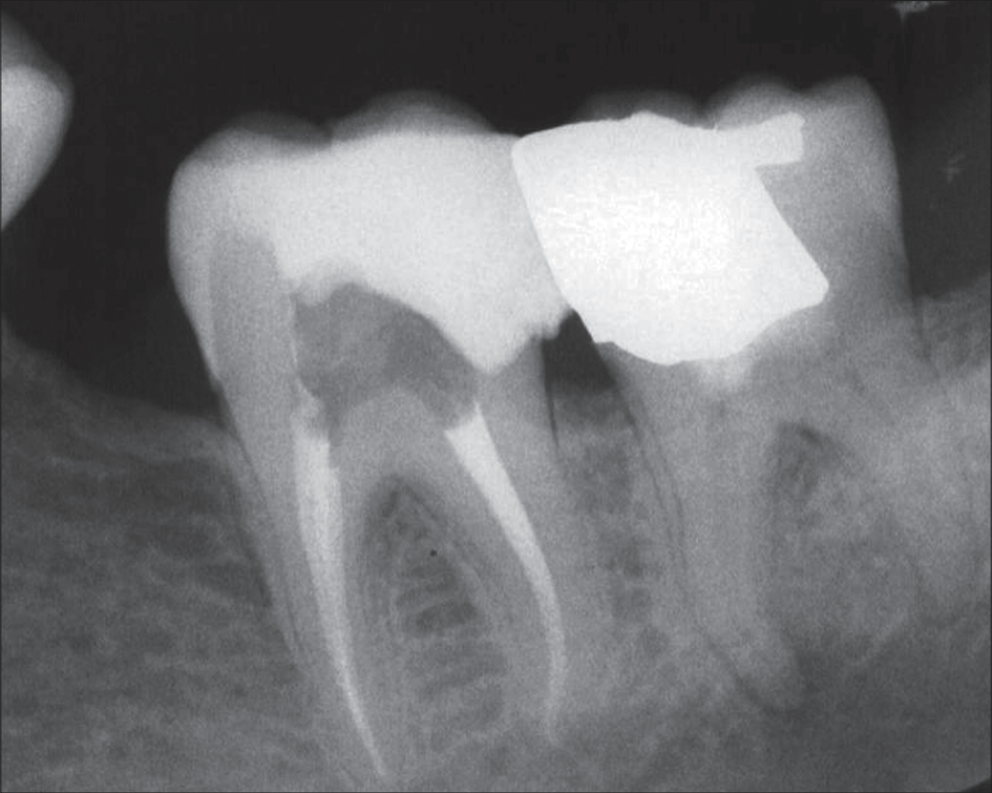
Immediate postoperative radiograph of the second molar after a week. Note the periradicular lesion in the mesial root of the third molar

**Figure 4:**
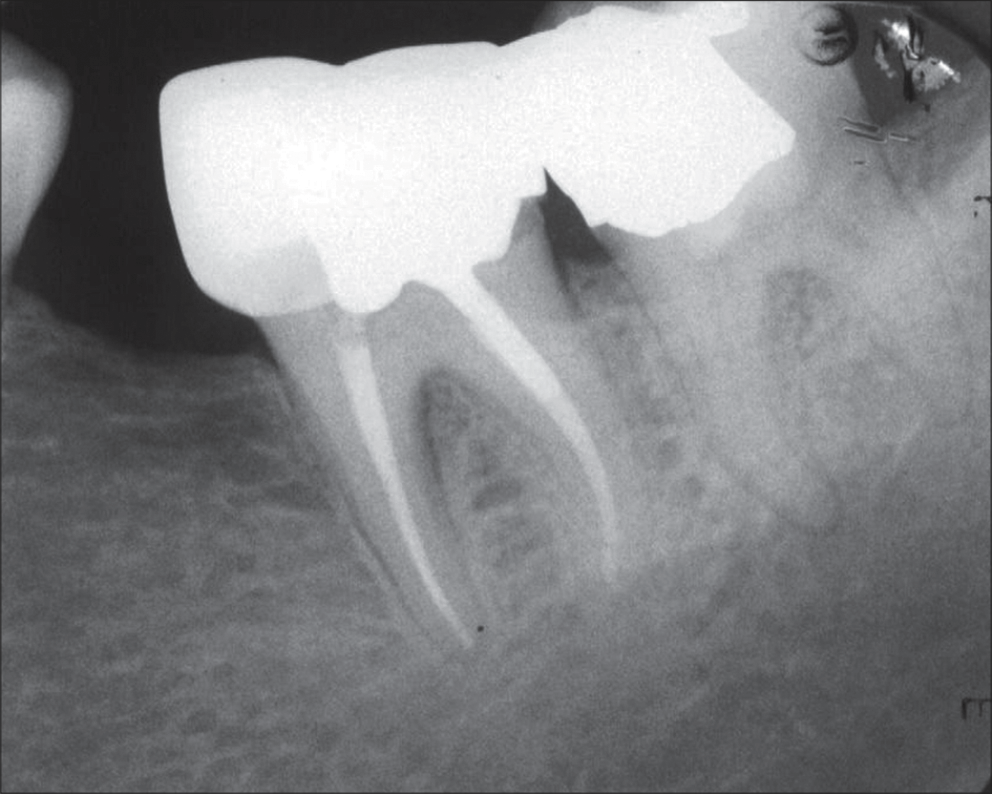
Twelve-month follow-up radiograph showing favorable outcomes. The treated teeth are in function and the periapical tissues are normal. An important finding is the complete healing of the periradicular lesion in the mesial root of the third molar

All patients (six males and six females aged 14 to 62) were available for recall. All cases had clinical diagnosis of irreversible pulpitis and there were no complaints of discomfort or tenderness at the first recall (+1 day). The mean time for next recall was 15.8 (13-20) months. At the next recall, tooth mobility was within the normal physiological range, percussion test was negative, and patients were asymptomatic. Radiographic examination showed normal periodontium apparatus [Figures 1–4].

**Figure 5:**
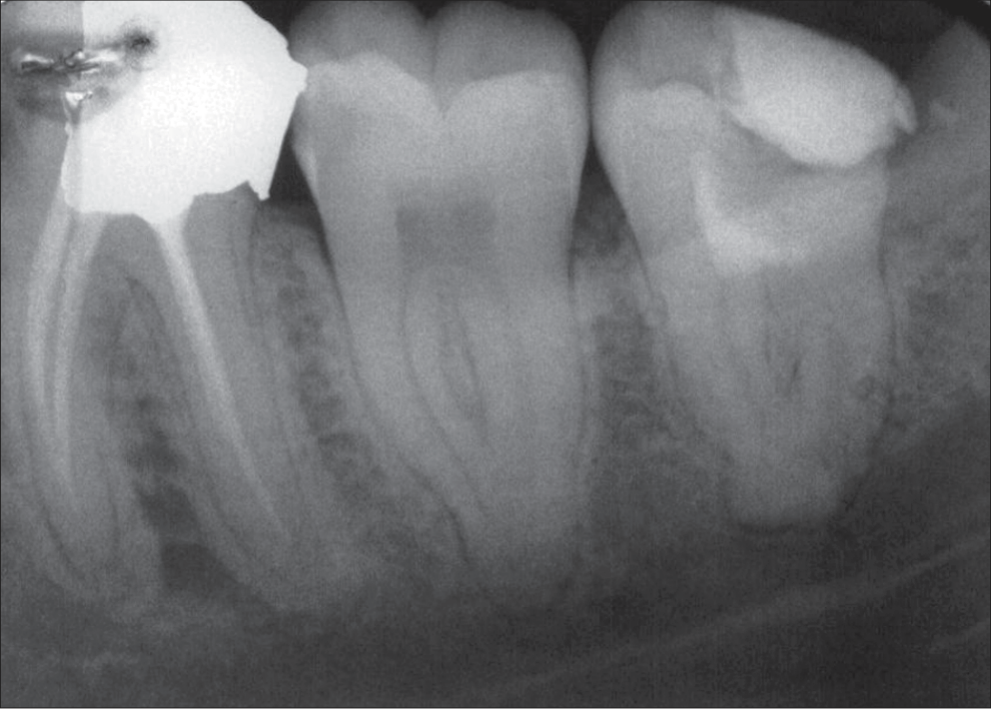
Postoperative radiograph showing pulpotomy with new endodontic material

**Figure 6:**
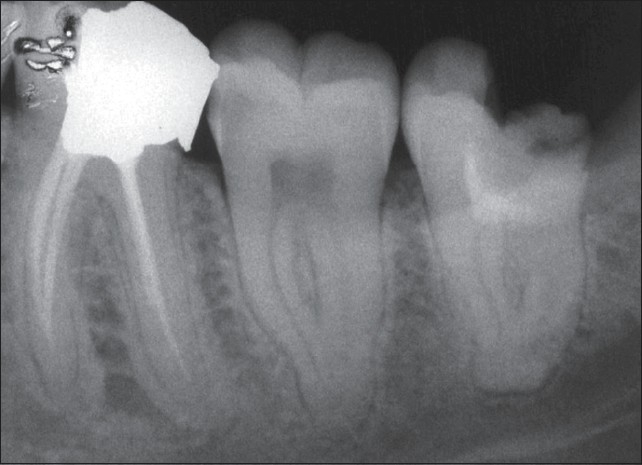
Two-month follow-up radiograph showing pulpotomized tooth without coronal filling. The patient had extracted the tooth because it was not restorable

**Figure 7:**
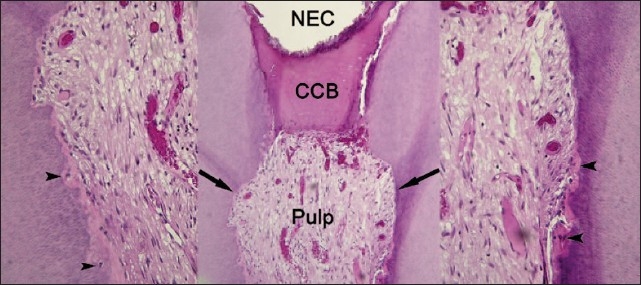
Center: a view of the pulpotomy area of a twomonth sample capped with NEC. There is no inflammatory reaction. A complete calcified bridge (CCB) was formed and normal pulpal tissue was completely excommunicated from the exterior. A few irregularities in the dentinal walls (arrows) indicated old internal resorption (H & E, ×100). Left and right: higher magnification of resorption areas (black arrows). A few cells being embedded (arrow heads) against resorption lacunae, demonstrating new predentin deposition and the end of the resorption process (H & E, ×400)

One of the teeth, a lower wisdom tooth, was extracted as direct restoration was deemed to be impractical and the patient refused to restore it prosthetically. The tooth was extracted after two months and histological assessment was performed. On microscopic examination, a complete calcified bridge was seen and there was no inflammation demonstrable in the pulpal tissue. Histological examination revealed evidence of old internal resorption. A few embedded cells and a layer of predentin was seen on the resorbed dentinal wall, implying arrested internal resorption [Figures 5–7].

## DISCUSSION

There are only few studies to evaluate pulpotomy in mature permanent teeth. Mc Dougal * et al*., performed eugenol pulpotomy for irreversible pulpitis in mature teeth.[5] Nyerere * et al., * carried out eugenol pulpotomy on adult patients with painful acute pulpitis,[12] and Nosrat and Nosrat performed CH pulpotomy on two adult teeth with carious exposures.[11] Eghbal * et al.′* s study also supports this argument. They demonstrated complete dentinal bridge formation and healthy pulps after treatment with MTA pulpotomy in mature permanent molars with irreversible pulpitis. Moreover, their success measure was based on the histological examination.[18] These studies provide a strong body of evidence that supports the clinical and radiographic results of the present study at the final recall sessions (13-20 months). Our results indicate that pulpotomy may be used as an alternative therapy for mature teeth with irreversible pulpitis. 

 Although normal aging of pulp reduces the chance of success in vital pulp therapy,[10] this study showed that the use of the appropriate materials will allow healing even in the aged pulp. 

Zilberman * et al*.,[23] in 1989 and Mejare and Cvek[24] in 1993, reported failure in vital pulp therapy, after 20 and 10 days, respectively. Nosrat and Nosrat concluded that the first few postoperative months after partial pulpotomy are critical; after that, the results remain constant.[11] We can therefore assume that 13-20 months are adequate. As the follow-up period in this study was sufficient to allow bacterial leakage and subsequent involvement of the radicular pulp to occur in failure cases[5] and since chronic apical periodontitis may be asymptomatic, periapical status was also evaluated radiographically. 

The favorable results in this study may be partly attributed to the immediate placement of permanent coronal restoration. As long-term failure is sometimes associated with microleakage caused by temporary restorative material, Massler demonstrated that the most important cause of failure in vital pulp therapy is the presence of leakage during the healing process.[25] 

It has been postulated that the existence of a hard tissue barrier may at least be an indicator of clinical success, and there are several studies that point to its clinical importance.[26] In the present study, one tooth was extracted due to a failed restoration. This case was microscopically examined and a complete calcific bridge was observed. The normal pulpal tissue was completely excommunicated from the external environment, which was critical for successful healing. There was also evidence of a previous internal resorbed dentin, with a succeeding layer of predentin, which indicated that internal resorption had ceased and dentin formation had occurred as a result of elimination of the inflammation. However, there are as yet no reports of internal resorption arresting after vital pulp therapy. 

Cox * et al.*[27] and Asgary * et al.,*[16] demonstrated the presence of tunnel defects in the dentinal barrier formed after CH pulp capping; these can serve as pathways for microleakage and pulp inflammation. The bridge under the NEC in the observed case had no irregularities or defects.

Ford suggested that bridge formation underneath the capping material could be due to its unique properties; such as, sealing ability, biocompatibility, alkalinity, or/and others.[14] Other studies have also suggested that the most important factor in the dental pulp healing process is the sealing ability of the material.[28] NEC prevents microleakage with a sealing ability superior to IRM and comparable to MTA.[22] Therefore, as a pulpotomy material, it can prevent recontamination of dental pulp, which can occur with CH compounds. It is also likely that in addition to its sealing ability, the antibacterial effect of the NEC[19] performed well in disinfecting the area, by disabling any bacteria that passed the barrier. A recent investigation has demonstrated the ability of NEC in actively stimulating hard tissue.[16] The biological mechanism by which NEC promotes calcific bridge formation is currently unknown. This characteristic is likely to be the result of several properties such as its sealing ability,[22] biocompatibility,[16] high alkalinity,[20] antibacterial effect[19] hydroxyapatite formation,[29] and similarity to dentine.[30] It is well accepted that the handling characteristics of clinically applied materials are of practical importance. Hydrophobic cements require a completely dry cavity, free of blood and saliva, NEC on the other hand is hydrophilic and requires a wet environment to set and therefore is ideal as a pulp capping material.[20] It appears that NEC meets the requirements for pulpotomy agents although a consensus on the exact requirements has not been formed. 

Mc Dougal * et al*., advocated pulpotomy as an interim form of management, as an alternative to extraction, for patients who wished to save teeth with irreversible pulpitis, but could not afford RCT at the time.[5] They recommended that the use of such a treatment should not exceed a period of 12 months,[5] although our study shows that pulpotomy can preserve teeth longer than 12 months. It appears that in their samples calcified bridge formation did not take place as the pulp was not in its natural environment and could not heal.

One of the selection criteria in this study was the clinical diagnosis of irreversible pulpitis, however, clinical findings could not exactly predict the histological status of the pulp. However, lingering pain might indicate severe pathosis of pulpal tissues. Previous studies emphasized that a clinical diagnosis of irreversible pulpitis is generally associated with moderate-to-severe changes of the pulpal tissue. Therefore, it was rational to assume that the pulps in this study were inflamed.[31] No inflammation was observed in the pulpal tissue after pulpotomy with NEC. It seems that the selection criteria in this study could create a commonly encountered clinical situation — cariously exposed teeth, complicated with irreversible pulpitis. As bacteria and their by-products could affect the pulpal response, this study may have more clinical relevance than others performed on healthy human teeth. 

Although, with limited number of subjects, we cannot conclude that pulpotomy is the treatment of choice in mature teeth, the advantages of keeping the tooth, saving time, and money are of great benefit to the patient.

## CONCLUSION

Based on the results of this case series as well as the properties of NEC, demonstrated in previous studies, it appears that NEC is suitable as a pulpotomy agent. The presence of a hard tissue bridge and normal pulp is a sign of a successful pulpotomy, which indicates regeneration. This is of clinical importance as coronal fillings may allow microleakage.

We can conclude that there is a reasonable biological argument to carry out pulpotomy as a possible alternative treatment in mature permanent teeth with irreversible pulpitis. Further studies can add significant weight to this argument.
